# Supplementary material for: Receptor‐mediated clustering of FIP200 bypasses the role of LC3 lipidation in autophagy
Source: EMBO J. 2020 Nov 23;39(24):e104948. doi: 10.15252/embj.2020104948 (PMC7737610; doi:10.15252/embj.2020104948)
Supplement: Supplementary file 1 — Appendix [file EMBJ-39-e104948-s001.pdf]

# APPENDIX

## Receptor-mediated clustering of FIP200 bypasses the role of LC3 lipidation in autophagy

**Authors: Amelia E Ohnstad[1], Jose M Delgado[1], Brian J North[1], Isha Nasa[1,2], Arminja N Kettenbach[1,2], Sebastian W. Schultz[3,4], Christopher J Shoemaker[1]***

**Affiliations:**

**[1] Department of Biochemistry and Cell Biology, Geisel School of Medicine at Dartmouth, Hanover, New Hampshire, USA**

**[2] Norris Cotton Cancer Center, Lebanon, NH, USA**
**[3] Centre for Cancer Cell Reprogramming, Faculty of Medicine, University of Oslo, Oslo, Norway**
**[4] Department of Molecular Cell Biology, Institute for Cancer Research, Oslo University Hospital, Oslo, Norway**

*** Christopher.J.Shoemaker@Dartmouth.edu (to C.J.S)**

**----------------------------------------------------**

**Appendix Figure Legend - Pages 1 & 2**
**Appendix Figure S1. - Page - 3**
**Appendix Figure S2 - Page - 4**
**----------------------------------------------------**

**<u>Appendix Figure Legend:</u>**

**Appendix Figure S1 – related to Fig 2.**

**(A)** Gating strategy for tf-Reporter screens. Single cells were selected through the indicated forward scatter and back scatter gates. Singlets were then analyzed for red and green fluorescence, with the indicated gates sorted to enrich for cells with activated and inhibited autophagy.

**(B)** A rank-ordered list of autophagy modifiers based on average beta scores across all lipidation deficient cell lines ($ATG7^{KO}$, $ATG10^{KO}$ and $ATG3^{KO}$ cells). Top modifiers of lipidation-deficient autophagy are indicated in blue. Data from Table EV2.

**(C)** Gene correlation plot of average beta scores for tf-NBR1 in wild-type cells (from (Shoemaker *et al*, 2019)) and $ATG7^{KO}$ cells (this study). Highlighted in red are genes with a beta score > 0.5 across all three lipidation-deficient cell lines. Dashed lines, top 1% of beta scores. Data from Table EV2.

**Appendix Figure S2 – related to Fig 6.**

**(A, B)** *ATG7*$^{KO}$/*TAX1BP1*$^{KO}$ K562 cells expressing tf-NBR1 were nucleofected with TagBFP-TAX1BP1 variants and analyzed for red:green ratio and BFP expression. (n = 10,000 cells for all samples in **A**, n>8,000 cells for all samples in **B**). Median values for each sample are identified by a black line within each violin. The red dotted line across all samples corresponds to the red:green ratio of rescued cells (i.e. TAX1BP1$^{WT}$). The red solid line across all samples corresponds to the ratio observed in non-transfected cells.

**(C, D)** *TAX1BP1*$^{KO}$ K562 cells were nucleofected with tf-TAX1BP1 variants and analyzed for red:green ratio (n = 10,000 cells for all *TAX1BP1*$^{KO}$ rescues. n>4,000 for *ATG9A*$^{KO}$ control). Median values for each sample are identified by a black line within each violin. The red dotted line across all samples corresponds to the red:green ratio of tf-TAX1BP1$^{WT}$. The red solid line across all samples corresponds to the red:green ratio observed for tf-TAX1BP1$^{WT}$ in *ATG9A*$^{KO}$ cells.

Source data are available online for this figure.

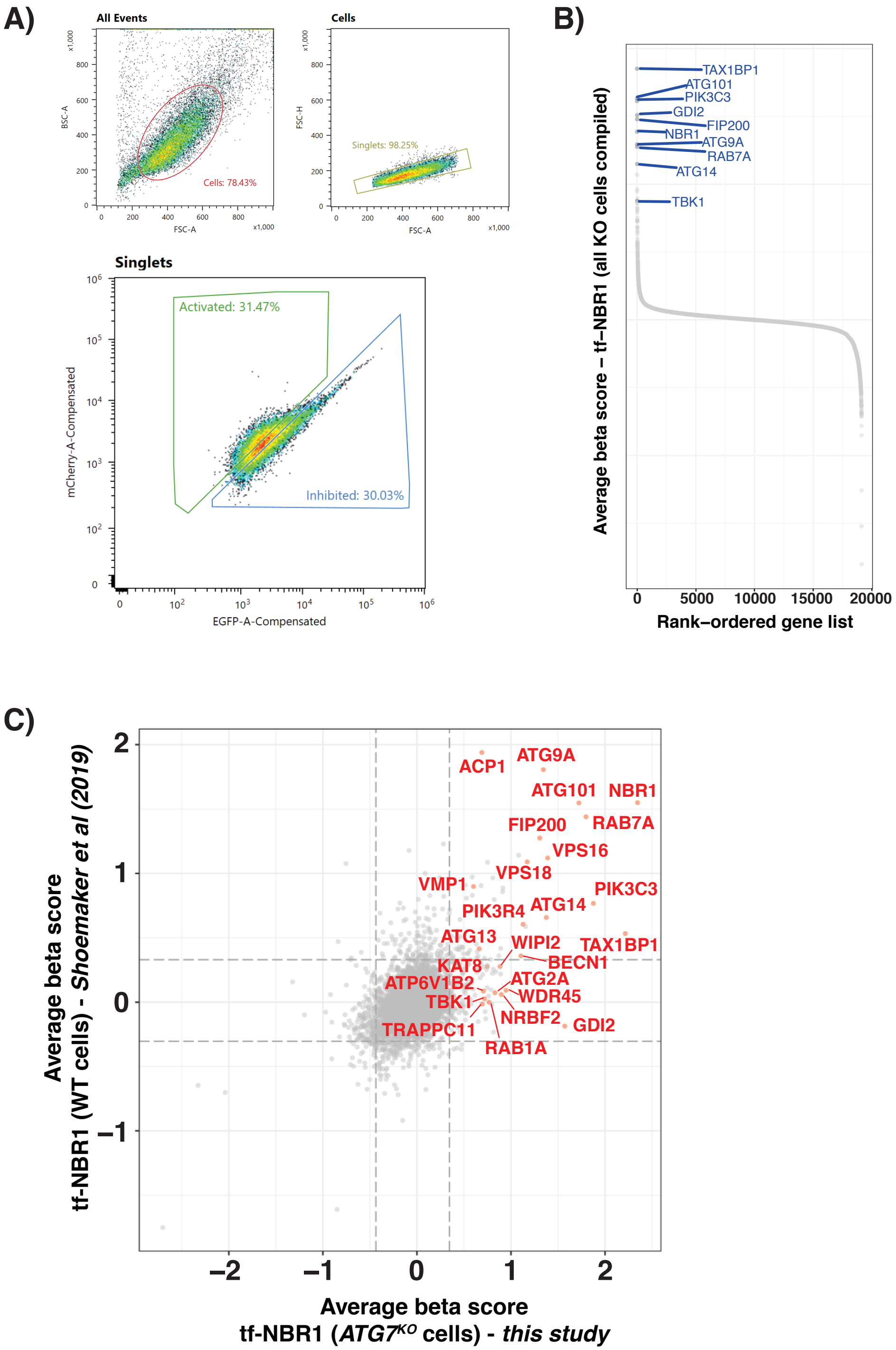


Appendix Figure S1

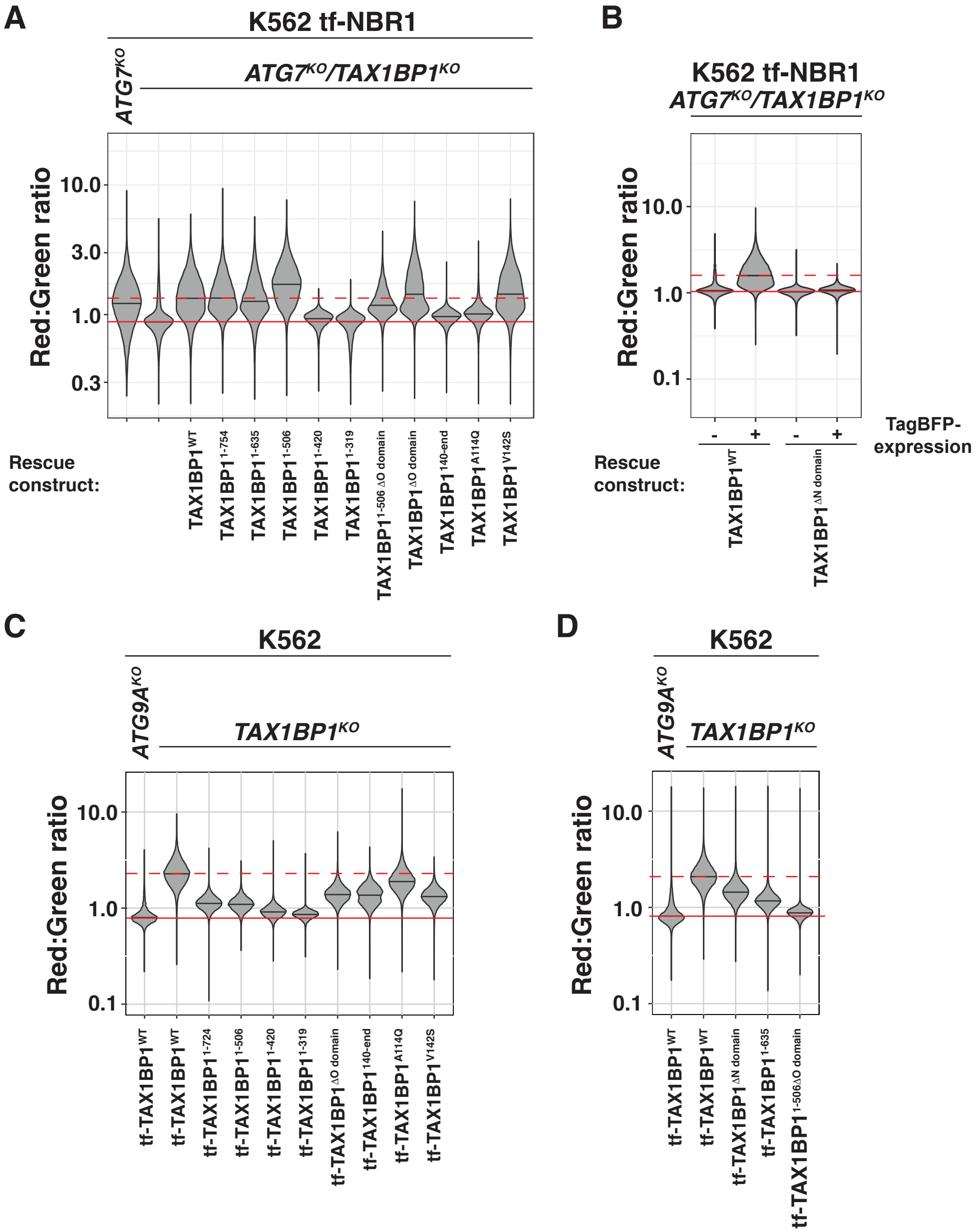

A
K562 tf-NBR1
ATG7KO
ATG7KO/TAX1BP1KO
Red:Green ratio
10.0
3.0
1.0
0.3
Rescue construct:
TAX1BP1WT
TAX1BP11-754
TAX1BP11-635
TAX1BP11-506
TAX1BP11-420
TAX1BP11-319
TAX1BP11-506 ΔO domain
TAX1BP1ΔO domain
TAX1BP1140-end
TAX1BP1A114Q
TAX1BP1V142S
B
K562 tf-NBR1
ATG7KO/TAX1BP1KO
Red:Green ratio
10.0
1.0
0.1
TagBFP-expression
- + - +
Rescue construct:
TAX1BP1WT
TAX1BP1ΔN domain
C
K562
ATG9AKO
TAX1BP1KO
Red:Green ratio
10.0
1.0
0.1
tf-TAX1BP1WT
tf-TAX1BP1WT
tf-TAX1BP11-724
tf-TAX1BP11-506
tf-TAX1BP11-420
tf-TAX1BP11-319
tf-TAX1BP1ΔO domain
tf-TAX1BP1140-end
tf-TAX1BP1A114Q
tf-TAX1BP1V142S
D
K562
ATG9AKO
TAX1BP1KO
Red:Green ratio
10.0
1.0
0.1
tf-TAX1BP1WT
tf-TAX1BP1WT
tf-TAX1BP1ΔN domain
tf-TAX1BP11-635
tf-TAX1BP11-506ΔO domain
