# Supplementary material for: Receptor‐mediated clustering of FIP200 bypasses the role of LC3 lipidation in autophagy
Source: EMBO J. 2020 Nov 23;39(24):e104948. doi: 10.15252/embj.2020104948 (PMC7737610; doi:10.15252/embj.2020104948)
Supplement: Supplementary file 2 — Expanded View Figures PDF [file EMBJ-39-e104948-s002.pdf]

Expanded View Figures

Figure EV1. NBR1 flux persists in lipidation-deficient cells (related to Fig 1).

A K562-derived extracts prepared from wild-type (WT) and clonal deletion isolates were resolved by SDS–PAGE followed by immunoblotting (IB) with indicated antibodies. All samples were normalized by total protein using a BCA assay prior to loading. I and II indicate the unmodified and lipidated forms of LC3, respectively.

B Wild-type and $ATG7^{KO}$ cells co-expressing Cas9 and tf-NBR1 were transduced with sgATG9A or a control sgRNA. After puromycin selection, cells were analyzed for red:green ratio by flow cytometry ($n > 10{,}000$ cells). Median values for each sample are identified by a black line within each violin.

C Representative confocal micrographs (as maximum intensity projections) of indicated K562 deletion cells expressing tf-NBR1. Selected regions (white box) of micrographs are shown as single and merged channels from fluorescence microscopy. Red fluorescent protein (RFP), magenta; Green fluorescent protein (GFP), green; Hoechst, blue. Scale bars: large panels, 5 μm; small panels, 1 μm. Related to Fig 1D.

D K562 wild-type (WT) and $FIP200^{KO}$ cells expressing tf-NBR1 or tf-LC3 were nucleofected with TagBFP or TagBFP-FIP200 and analyzed for red:green ratio and BFP expression at 8 h post-nucleofection. Median values for each sample are identified by a black line within each violin. The red dotted line corresponds to the red:green ratio of parental cells expressing BFP-FIP200. The black dotted line corresponds to the ratio observed under maximally inhibited conditions ($FIP200^{KO}$, non-rescued). NBR, $n > 750$ cells; LC3, $n > 1{,}500$ cells.

E Correlative light and electron microscopy (CLEM) of K562 $ATG7^{KO}$ cells expressing tf-NBR1 under basal conditions. Analysis workflow is indicated by green arrows. White boxes demarcate zoomed area in subsequent images. NBR1, green; Hoechst, blue. Scale bar (small images), 2.5 μm. Scale bar (large images), 250 nm. Related to Fig 1E.

F Plot showing fractional turnover of NBR1 as a function NBR1 expression level. Wild-type and deletion cells were transfected with a CMV-driven tf-NBR1 construct and cells were monitored for RFP and GFP fluorescence. Fractional turnover was calculated as $(RFP^{intensity} - GFP^{intensity})/(RFP^{intensity} - GFP^{background})$. Non-fluorescent cells (log[RFP] < 0.85) were excluded from the plot. $n > 12{,}000$ cells per condition.

Source data are available online for this figure.

© 2020 The Authors

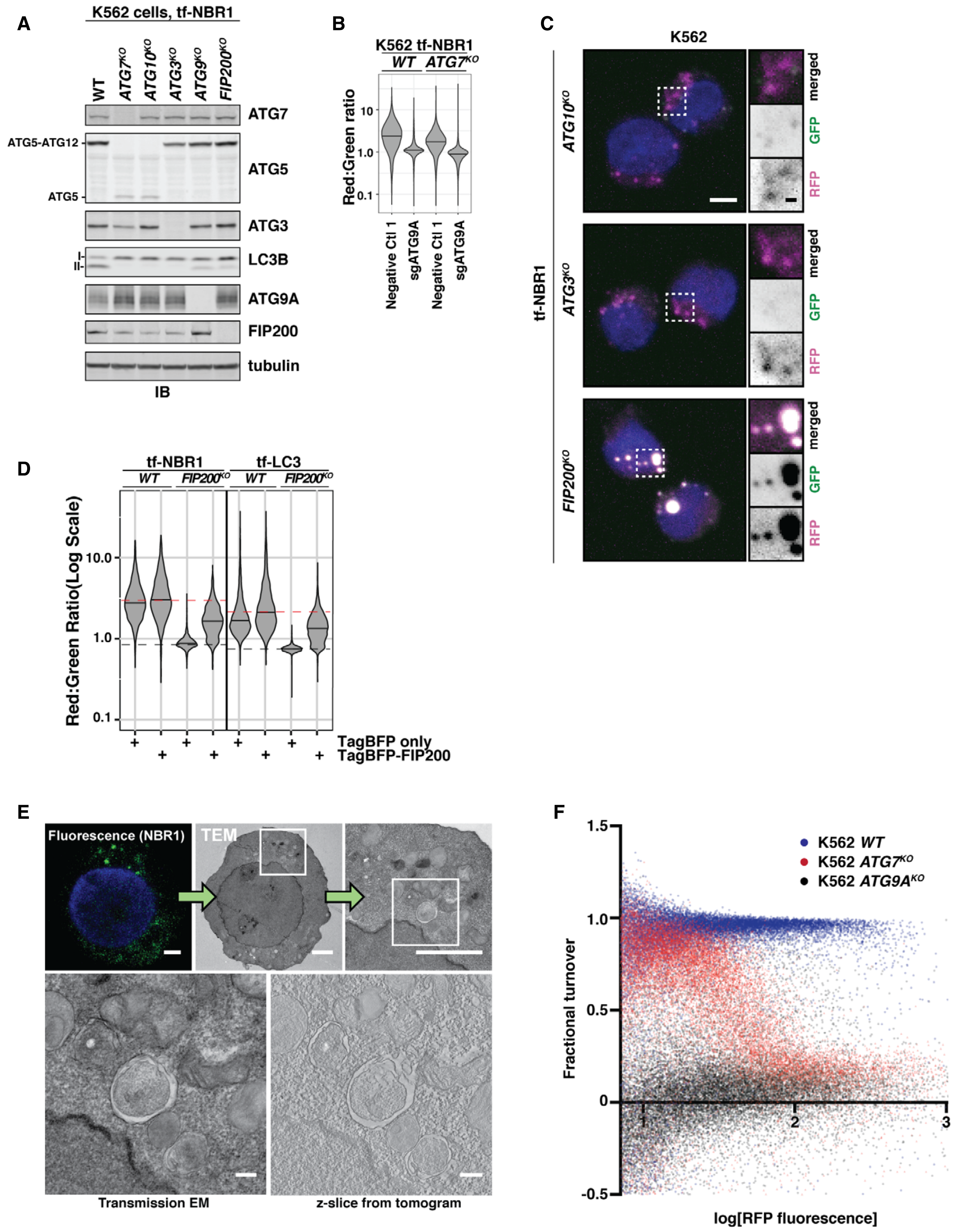

Figure EV1.

© 2020 The Authors

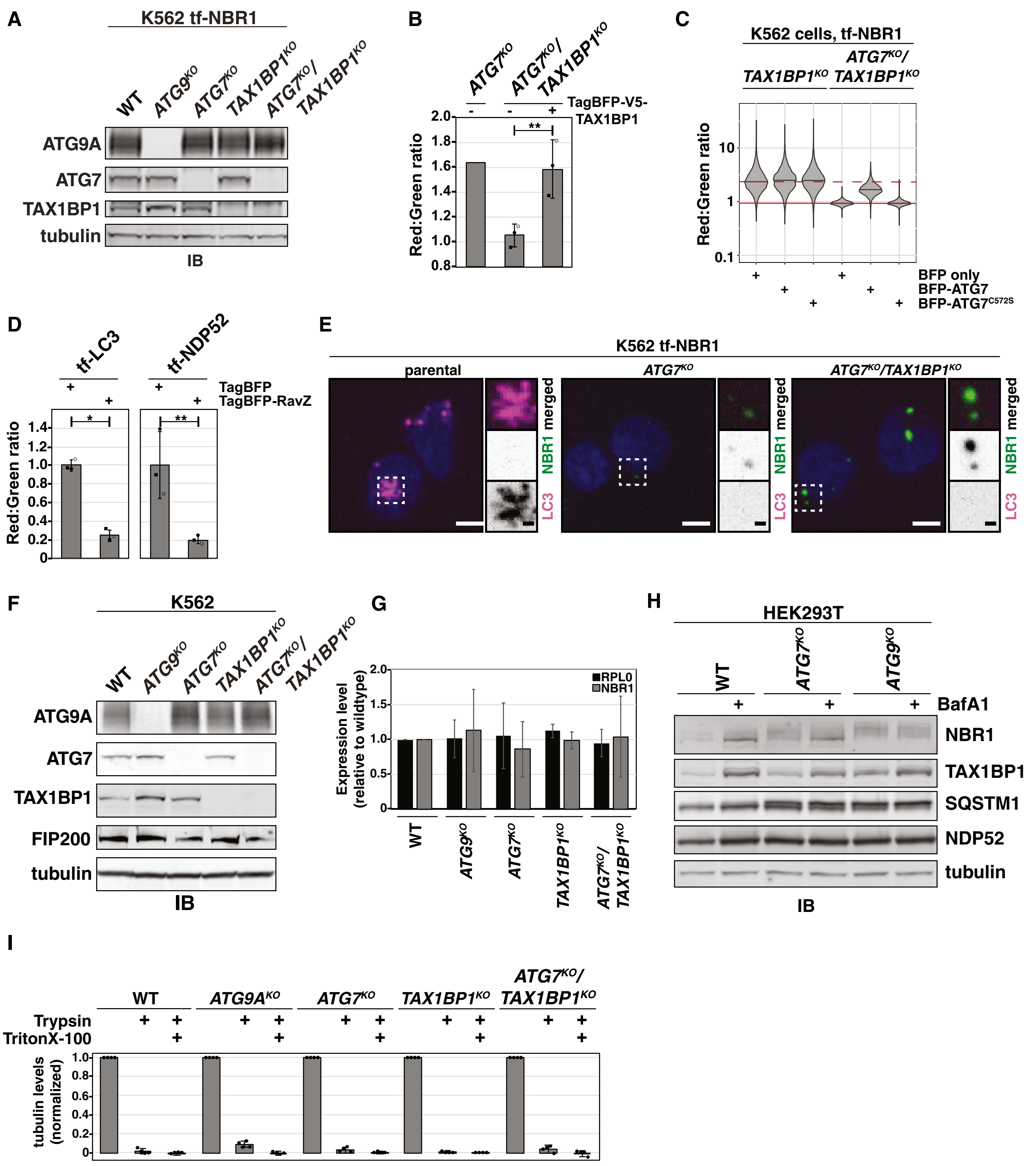
A
K562 tf-NBR1
WT
ATG9KO
ATG7KO
TAX1BP1KO
ATG7KO/TAX1BP1KO
ATG9A
ATG7
TAX1BP1
tubulin
IB
B
ATG7KO
ATG7KO/TAX1BP1KO
TagBFP-V5-TAX1BP1
Red:Green ratio
C
K562 cells, tf-NBR1
TAX1BP1KO
ATG7KO/TAX1BP1KO
Red:Green ratio
BFP only
BFP-ATG7
BFP-ATG7C572S
D
tf-LC3
tf-NDP52
TagBFP
TagBFP-RavZ
Red:Green ratio
E
K562 tf-NBR1
parental
ATG7KO
ATG7KO/TAX1BP1KO
merged
NBR1
LC3
F
K562
WT
ATG9KO
ATG7KO
TAX1BP1KO
ATG7KO/TAX1BP1KO
ATG9A
ATG7
TAX1BP1
FIP200
tubulin
IB
G
RPL0
NBR1
Expression level (relative to wildtype)
WT
ATG9KO
ATG7KO
TAX1BP1KO
ATG7KO/TAX1BP1KO
H
HEK293T
WT
ATG7KO
ATG9KO
BafA1
NBR1
TAX1BP1
SQSTM1
NDP52
tubulin
IB
I
WT
ATG9AKO
ATG7KO
TAX1BP1KO
ATG7KO/TAX1BP1KO
Trypsin
TritonX-100
tubulin levels (normalized)

Figure EV2.

Figure EV2. TAX1BP1 is required for NBR1 flux in lipidation-deficient cells (related to Figs 3 and 4).

A K562-derived extracts prepared from wild-type (WT) and clonal deletion isolates were resolved by SDS–PAGE followed by immunoblotting (IB) with indicated antibodies. All samples were normalized by total protein using a BCA assay prior to loading.
B *ATG7*^{KO}/*TAX1BP1*^{KO} cells expressing tf-NBR1 were transfected with TagBFP-V5-TAX1BP1 and analyzed for red:green ratio and BFP expression. Plotted are the median red:green ratios of BFP-negative and BFP-positive cells relative to *ATG7*^{KO} cells alone. Bar graphs represent mean ± SD from three independently generated *ATG7*^{KO}/*TAX1BP1*^{KO} cell lines. *P* values were determined using a ratio paired *t*-test. $^{**}P < 0.01$; ns, not significant. $n = 10{,}000$ cells per sample.
C *TAX1BP1*^{KO} and *ATG7*^{KO}/*TAX1BP1*^{KO} K562 cells expressing tf-NBR1 were nucleofected with TagBFP, TagBFP-ATG7, or TagBFP-ATG7^{C572S} and analyzed for red:green ratio and BFP expression at 8 h post-nucleofection. Median values for each sample are identified by a black line within each violin. The red dotted line corresponds to the red:green ratio of *TAX1BP1*^{KO} cells expressing TagBFP. The solid red line corresponds to the red:green ratio of *ATG7*^{KO}/*TAX1BP1*^{KO} cells expressing TagBFP. $n > 5{,}000$ cells for each sample.
D Wild-type K562 cells expressing tf-LC3 or tf-NDP52 were transfected with TagBFP or TagBFP-RavZ. BFP-positive cells were analyzed for red:green ratio. The median red:green ratio of each sample was used to calculate flux relative to WT + TagBFP. Bar graphs represent mean ± SD from three independent experiments. $n > 10{,}000$ cells per sample. *P* values were determined using a ratio paired *t*-test. $^{*}P < 0.05$; $^{**}P < 0.005$; ns, not significant.
E Representative confocal micrographs (as maximum intensity projections) of indicated K562 genotypes expressing tf-NBR1. Selected regions (white box) of micrographs are shown as single and merged channels from fluorescence microscopy. LC3, magenta; NBR1, green; Hoechst, blue. Scale bars: large panels, 5 μm; small panels, 1 μm.
F K562-derived extracts prepared from wild-type (WT) and clonal deletion isolates were resolved by SDS–PAGE followed by immunoblotting (IB) with indicated antibodies. All samples were normalized by total protein using a BCA assay prior to loading.
G Total RNA was extracted from the indicated cell lines. Equal amounts of subsequently generated cDNA were analyzed for NBR1 expression levels. All values were normalized to GAPDH expression levels using the ΔΔC method. RPL0 was included as an unchanged control. Bar graphs represent mean ± SD. $n = 3$ technical replicates.
H HEK293T cells with indicated genotypes were treated with Bafilomycin A1 (BafA1) or DMSO for 18h. Extracts prepared from wild-type (WT) and deletion cell lines were resolved by SDS–PAGE followed by immunoblotting (IB) with indicated antibodies. All samples were normalized by total protein using a BCA assay prior to loading.
I Quantitation of protease protection data for tubulin from experiments in Fig 4E. Bar graphs show the mean ± SD of each sample from four independent experiments.

Source data are available online for this figure.

Figure EV3. Autophagy receptor dynamics in ATG7-ndependent autophagy (related to Fig 5).

A Extracts derived from K562 cells with or without integrated tf-TAX1BP1 were normalized for total protein by BCA. TAX1BP1 was immunoprecipitated using GFP-Trap dynabeads. Input and eluate were resolved by SDS–PAGE followed by immunoblotting (IB) with indicated antibodies.
B Extracts derived from *ATG9A*^{KO} K562 cells with or without integrated tf-NBR1 were normalized for total protein by BCA. NBR1 was immunoprecipitated using GFP-Trap dynabeads. Input and eluate were resolved by SDS–PAGE followed by immunoblotting (IB) with indicated antibodies.
C K562-derived extracts prepared from wild-type (WT) and clonal deletion isolates were resolved by SDS–PAGE followed by immunoblotting (IB) with indicated antibodies. All samples were normalized by total protein using a BCA assay prior to loading.
D K562 cells co-expressing Cas9 and tf-NDP52 or tf-TAX1BP1 were transduced with sgATG9A or control sgRNAs. After 8 days of puromycin selection, K562-derived extracts were prepared and resolved by SDS–PAGE followed by immunoblotting (IB) with indicated antibodies. All samples were normalized by total protein using a BCA assay prior to loading.
E K562 cells from (D) were analyzed for red:green ratio by flow cytometry ($n = 10{,}000$ cells for each sample). Median values for each sample are identified by a black line within each violin.
F Wild-type and *ATG7*^{KO} cells co-expressing Cas9 and tf-NBR1 were transduced with sgSQSTM1 or a non-targeting control (sgControl). After 8 days of puromycin selection, K562-derived extracts were prepared and resolved by SDS–PAGE followed by immunoblotting (IB) with indicated antibodies. All samples were normalized by total protein using a BCA assay prior to loading.
G K562 cells from (F) were analyzed for red:green ratio by flow cytometry. Median values for each sample are identified by a black line within each violin. The red dotted line corresponds to the red:green ratio of tf-NBR1 in WT sgControl cells. The black dotted line corresponds to the ratio observed upon Bafilomycin A1 (BafA1) treatment (autophagy-dead). $n = 10{,}000$ cells for each sample.
H Wild-type K562 cells expressing tf-NBR1 were nucleofected with TagBFP or TagBFP-SQSTM1 and analyzed for red:green ratio and BFP expression at 18 h post-nucleofection. Median values for each sample are identified by a black line within each violin. The red dotted line corresponds to the red:green ratio of tf-NBR1 in WT, BFP-only cells. The black dotted line corresponds to the ratio observed upon Bafilomycin A1 (BafA1) treatment (autophagy-dead). $n = 10{,}000$ cells for each sample.
I Wild-type K562 cells expressing tf-SQSTM1 were nucleofected with TagBFP or TagBFP-NBR1 and analyzed for red:green ratio and BFP expression at 18 h post-nucleofection. Median values for each sample are identified by a black line within each violin. The red dotted line corresponds to the red:green ratio of tf-SQSTM1 in BFP-negative cells. The black dotted line corresponds to the ratio observed upon Bafilomycin A1 (BafA1) treatment (autophagy-dead). $n > 2{,}000$ cells for each sample.

Source data are available online for this figure.

© 2020 The Authors

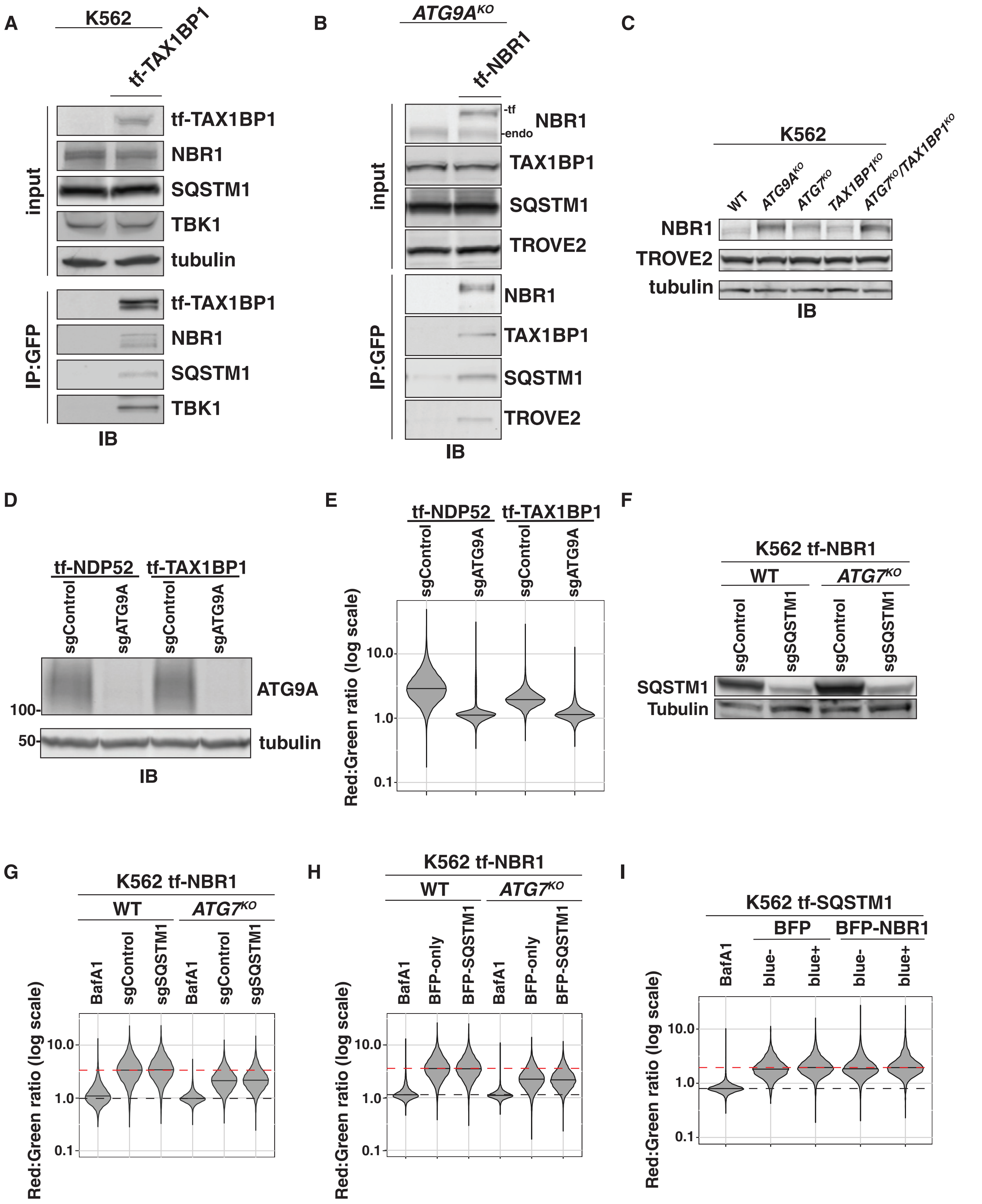
A
K562
tf-TAX1BP1
input
IP:GFP
tf-TAX1BP1
NBR1
SQSTM1
TBK1
tubulin
tf-TAX1BP1
NBR1
SQSTM1
TBK1
IB
B
ATG9AKO
tf-NBR1
-tf
-endo
NBR1
TAX1BP1
SQSTM1
TROVE2
NBR1
TAX1BP1
SQSTM1
TROVE2
IB
C
K562
WT
ATG9AKO
ATG7KO
TAX1BP1KO
ATG7KO/TAX1BP1KO
NBR1
TROVE2
tubulin
IB
D
tf-NDP52
tf-TAX1BP1
sgControl
sgATG9A
sgControl
sgATG9A
ATG9A
100-
50-
tubulin
IB
E
tf-NDP52
tf-TAX1BP1
sgControl
sgATG9A
sgControl
sgATG9A
Red:Green ratio (log scale)
10.0
1.0
0.1
F
K562 tf-NBR1
WT
ATG7KO
sgControl
sgSQSTM1
sgControl
sgSQSTM1
SQSTM1
Tubulin
G
K562 tf-NBR1
WT
ATG7KO
BafA1
sgControl
sgSQSTM1
BafA1
sgControl
sgSQSTM1
Red:Green ratio (log scale)
10.0
1.0
0.1
H
K562 tf-NBR1
WT
ATG7KO
BafA1
BFP-only
BFP-SQSTM1
BafA1
BFP-only
BFP-SQSTM1
Red:Green ratio (log scale)
10.0
1.0
0.1
I
K562 tf-SQSTM1
BFP
BFP-NBR1
BafA1
blue-
blue+
blue-
blue+
Red:Green ratio (log scale)
10.0
1.0
0.1

Figure EV3.

Figure EV4. The N-terminal SKICH domain and NBR1-binding domain of TAX1BP1 are required for ATG7-independent flux of NBR1 (related to Fig 6).

A HEK293T cells were transfected with myc-TAX1BP1^{WT} and indicated TagBFP-V5-TAX1BP1 variants. Extracts derived from transfected cells were immunoprecipitated (IP) with anti-TagBFP-conjugated dynabeads. Input and eluates were resolved by SDS–PAGE followed by immunoblotting (IB) with indicated antibodies.

B Representative confocal micrographs (as maximum intensity projections) of wild-type K562 cells and indicated deletion cells expressing tf-NBR1. Selected regions (white box) of micrographs are shown as single and merged channels from fluorescence and immunofluorescence microscopy against indicated proteins. Ubiquitin, magenta; NBR1, green; Hoechst, blue. Scale bars: large panels, 5 μm; small panels, 1 μm.

C Wild-type K562 cells were nucleofected with tf-NBR1 (isoform 1) or tf-NBR1 (isoform 2; lacking the UBA domain) and analyzed for red:green ratio at 18 h post-nucleofection. Median values for each sample are identified by a black line within each violin. The red dotted line (max value) corresponds to the red:green ratio of tf-NBR1 isoform 1. The solid red line (min value) corresponds to the red:green ratio of tf-NBR1 isoform 1 upon Bafilomycin A1 (BafA1) treatment. $n > 2{,}000$ cells for each sample.

D Representative confocal micrographs (as maximum intensity projections) of *ATG9A*^{KO}/*TAX1BP1*^{KO} cells expressing tf-NBR1. Cells were transduced with the indicated BFP-tagged TAX1BP1 variants. Selected regions (white box) of micrographs are shown as single and merged channels from fluorescence microscopy. BFP-TAX1BP1, magenta; NBR1, green; Scale bars: large panels, 5 μm; small panels, 1 μm.

E K562-derived extracts prepared from wild-type (WT) and clonal deletion isolates were resolved by SDS–PAGE followed by immunoblotting (IB) with indicated antibodies. All samples were normalized by total protein using a BCA assay prior to loading.

F Clonal isolates of indicated knockout cells lines (from (E)) were treated with Bafilomycin A1 (BafA1) or DMSO for 18 h and analyzed for tf-NBR1 flux by red:green ratio as assessed by flow cytometry. Median values for each sample are identified by a black line within each violin. The red dotted line across all samples corresponds to the red:green ratio of WT cells (normal flux). The red solid line across all samples corresponds to the ratio observed under maximally inhibited conditions (*ATG9A*^{KO} cells). $n > 5{,}000$ cells.

G HEK293T cells were transfected with indicated TagBFP-V5-TAX1BP1 variants. Extracts derived from transfected cells were immunoprecipitated (IP) with anti-TagBFP conjugated dynabeads. Input and eluates were resolved by SDS–PAGE followed by immunoblotting (IB) with indicated antibodies.

H HEK293T cells were transfected with HA-FIP200 and indicated TagBFP-V5-TAX1BP1 variants. Extracts derived from transfected cells were immunoprecipitated (IP) with anti-TagBFP conjugated dynabeads. Input and eluates were resolved by SDS–PAGE followed by immunoblotting (IB) with indicated antibodies.

Source data are available online for this figure.

 © 2020 The Authors

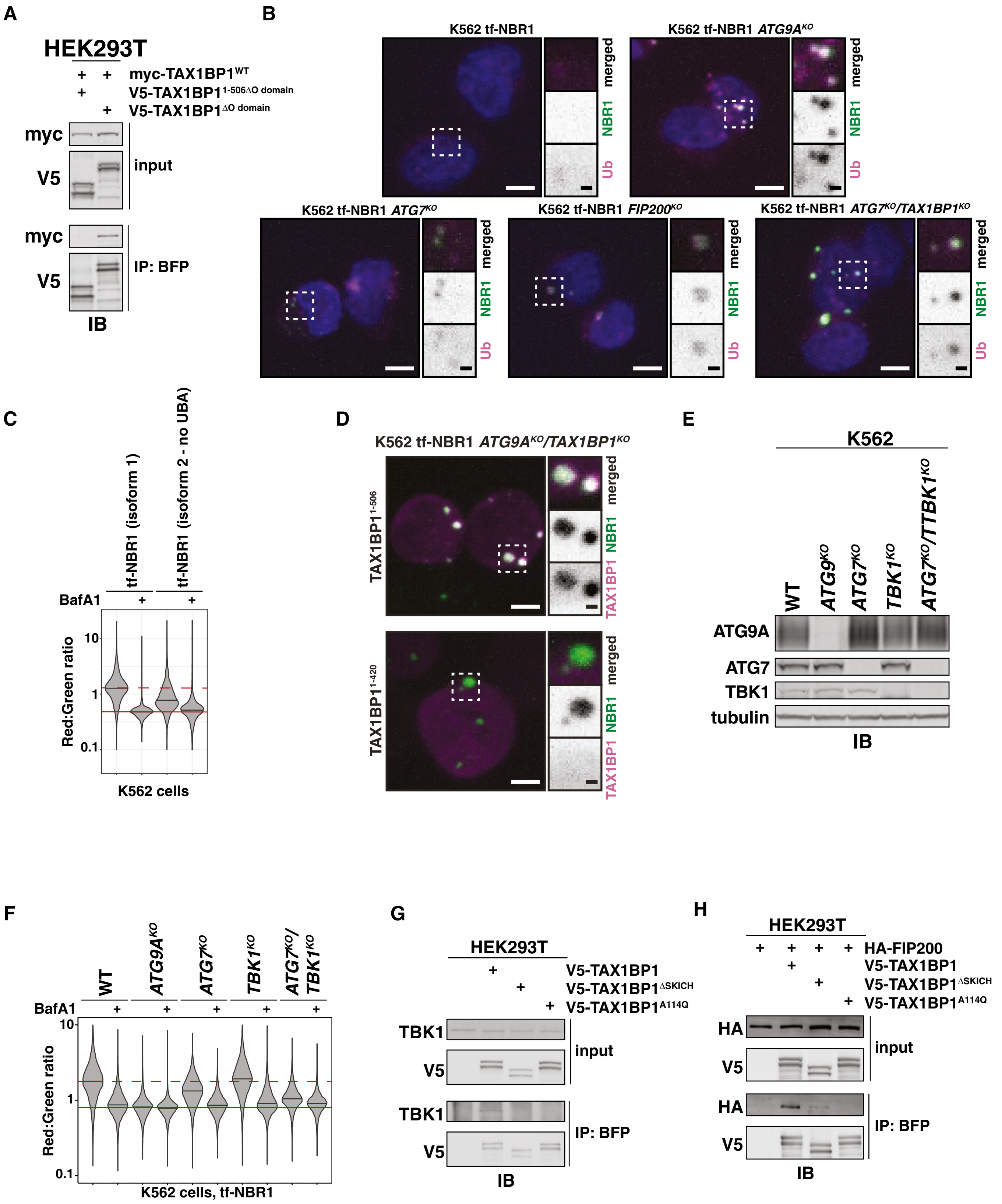
A
HEK293T
myc-TAX1BP1WT
V5-TAX1BP1 1-506ΔO domain
V5-TAX1BP1 ΔO domain
myc
V5
input
myc
V5
IP: BFP
IB
B
K562 tf-NBR1
K562 tf-NBR1 ATG9AKO
K562 tf-NBR1 ATG7KO
K562 tf-NBR1 FIP200KO
K562 tf-NBR1 ATG7KO/TAX1BP1KO
merged
NBR1
Ub
C
tf-NBR1 (isoform 1)
tf-NBR1 (isoform 2 - no UBA)
BafA1
Red:Green ratio
K562 cells
D
K562 tf-NBR1 ATG9AKO/TAX1BP1KO
TAX1BP1 1-506
TAX1BP1 1-420
merged
NBR1
TAX1BP1
E
K562
WT
ATG9KO
ATG7KO
TBK1KO
ATG7KO/TTBK1KO
ATG9A
ATG7
TBK1
tubulin
IB
F
WT
ATG9AKO
ATG7KO
TBK1KO
ATG7KO/ TBK1KO
BafA1
Red:Green ratio
K562 cells, tf-NBR1
G
HEK293T
V5-TAX1BP1
V5-TAX1BP1ΔSKICH
V5-TAX1BP1A114Q
TBK1
V5
input
TBK1
V5
IP: BFP
IB
H
HEK293T
HA-FIP200
V5-TAX1BP1
V5-TAX1BP1ΔSKICH
V5-TAX1BP1A114Q
HA
V5
input
HA
V5
IP: BFP
IB

Figure EV4.

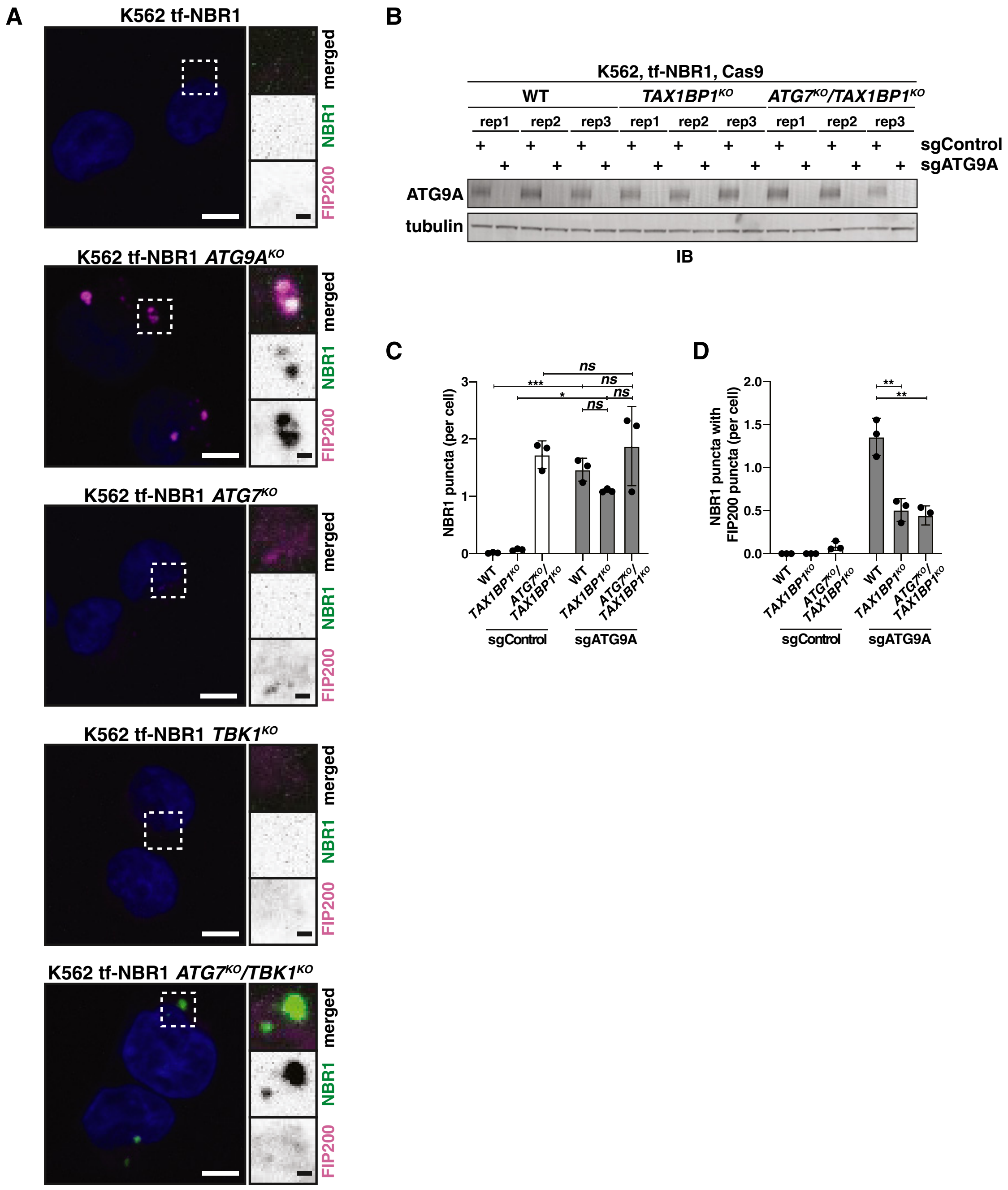

Figure EV5.

 © 2020 The Authors

◀ **Figure EV5. TAX1BP1-mediated clustering of FIP200 enables ATG7-independent flux of NBR1 (related to Fig 7).**

A Representative confocal micrographs (as maximum intensity projections) from wild-type and deletion K562 cells expressing tf-NBR1. Selected regions (white box) of micrographs are shown as insets of single and merged channels from fluorescence and immunofluorescence microscopy against indicated proteins. FIP200, magenta; NBR1, green; Hoechst, blue. Scale bars: large panels, 5 μm; small panels, 1 μm.

B Wild-type (WT) and indicated deletion isolates of K562 cells co-expressing tf-NBR1 and Cas9 were transduced with sgATG9A or a control sgRNA. After 8 days of puromycin selection, K562-derived extracts were prepared and resolved by SDS–PAGE followed by immunoblotting (IB) with indicated antibodies. All samples were normalized by total protein using a BCA assay prior to loading.

C Plots showing quantitation of NBR1 puncta per cell in wild-type, $TAX1BP1^{KO}$, and $ATG7^{KO}/TAX1BP1^{KO}$ cells imaged in Fig 7C (see Materials and Methods for details of quantitation). Bar graphs represent mean ± SD for three independently generated deletion cell lines (dots). $n > 150$ cells for each biological replicate. Samples were compared using a one-way ANOVA ($P < 0.0001$) with Tukey's HSD post-test. ***$P = 0.001$. *$P < 0.05$; ns, not significant.

D Plot showing quantitation of FIP200-positive NBR1 puncta per cell in wild-type, $TAX1BP1^{KO}$, and $ATG7^{KO}/TAX1BP1^{KO}$ cells imaged in Fig 7C. Bar graphs represent mean ± SD for three independently generated deletion cell lines (dots). $n > 150$ cells for each biological replicate. sgATG9A samples were compared using a one-way ANOVA ($P < 0.0001$) with Tukey's HSD post-test. **$P < 0.005$. a.u., arbitrary units.

Source data are available online for this figure.

© 2020 The Authors
