# Supplementary figures and images for: Receptor‐mediated clustering of FIP200 bypasses the role of LC3 lipidation in autophagy
Source: EMBO J. 2020 Nov 23;39(24):e104948. doi: 10.15252/embj.2020104948 (PMC7737610; doi:10.15252/embj.2020104948)

# Source data EV5

**B**

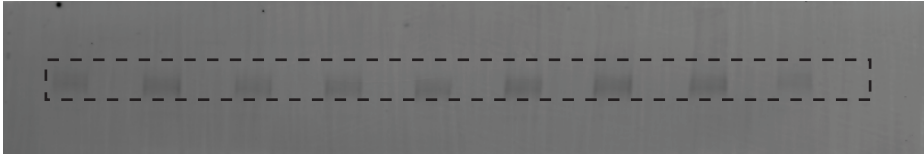

**ATG9A**

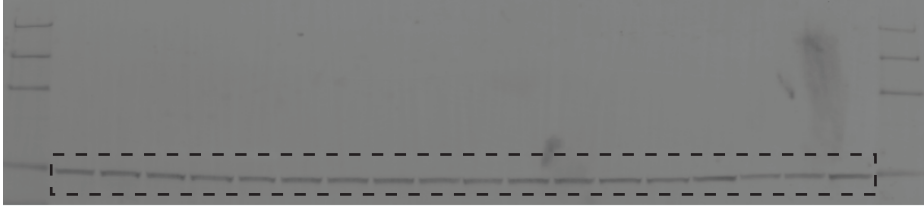

**Tubulin**

Supplement: Supplementary file 6 — Source Data for Expanded View [file EMBJ-39-e104948-s010.zip › EMBOJ-2020-104948R_SourceDataEV5.pdf]

# Source data EV4

A

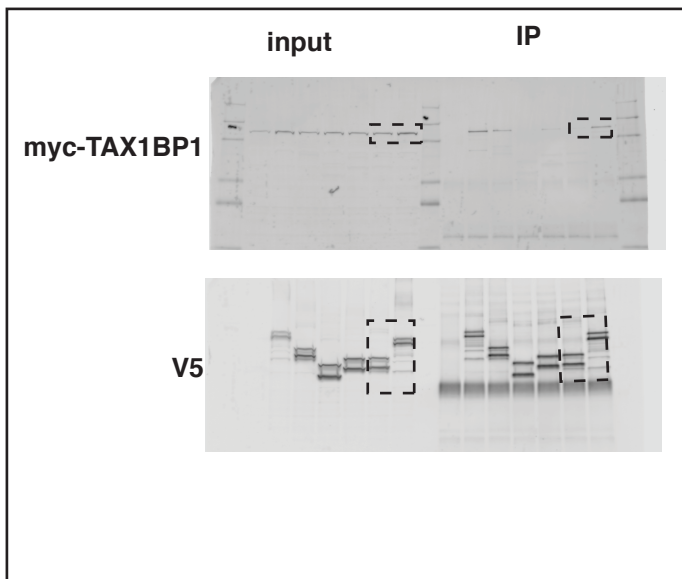

E

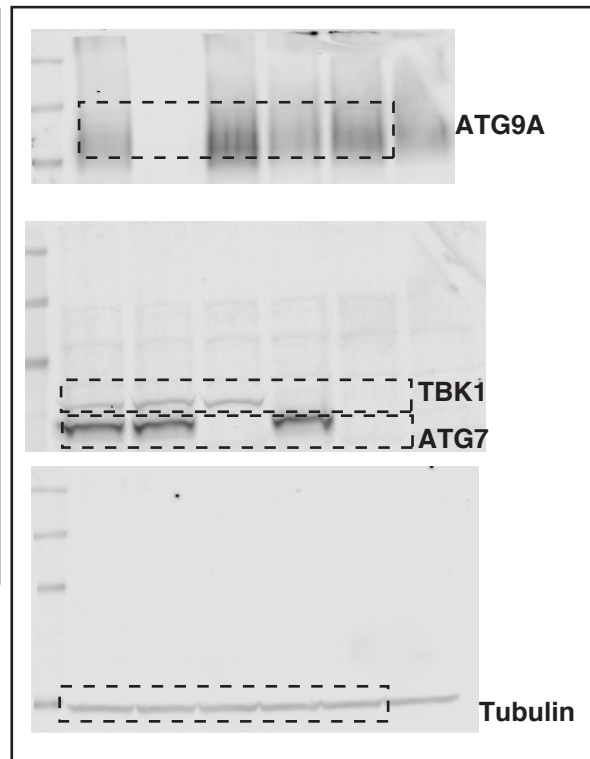

G

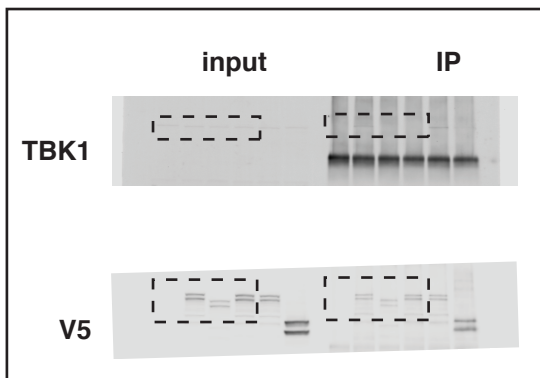

H

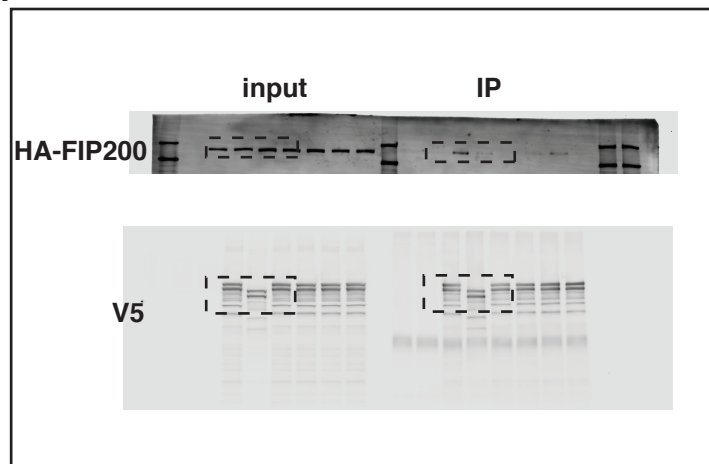

Supplement: Supplementary file 6 — Source Data for Expanded View [file EMBJ-39-e104948-s010.zip › EMBOJ-2020-104948R_SourceDataEV4.pdf]

# Source data EV3

**A**

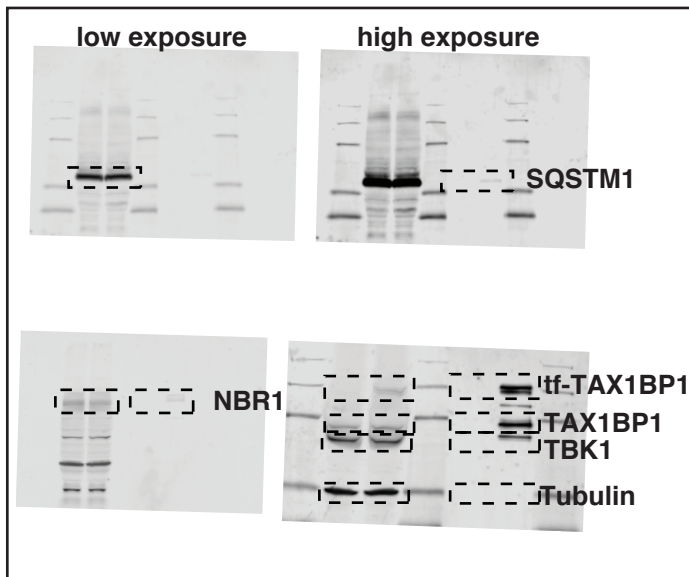

**C**

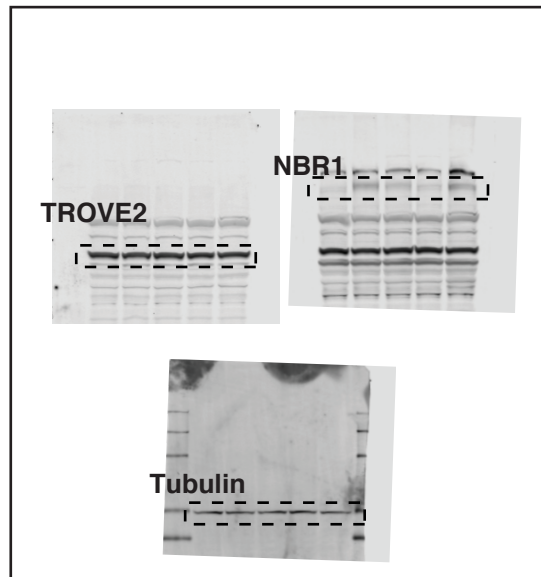

**B**

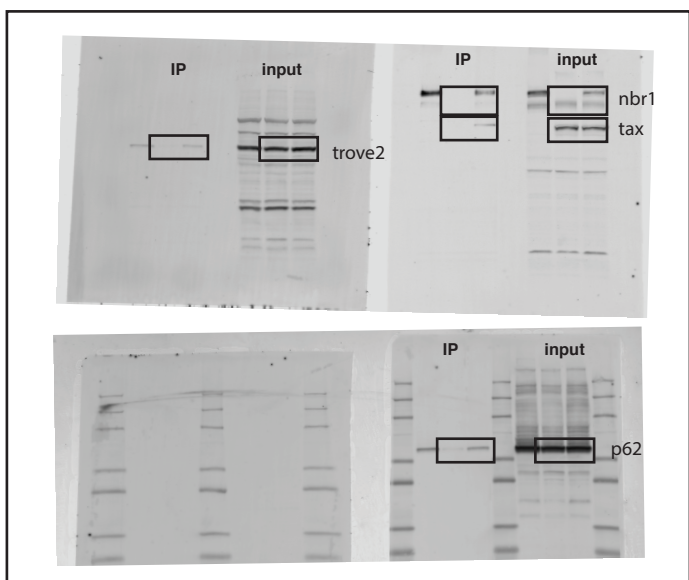

**D**

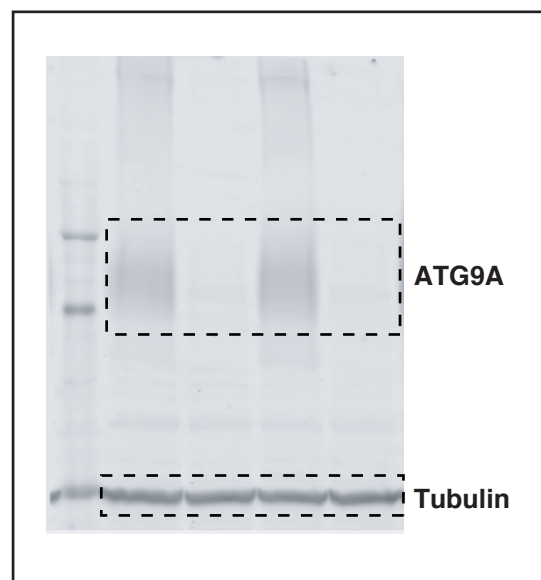

**F**

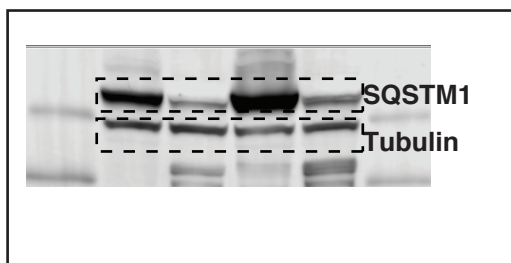

Supplement: Supplementary file 6 — Source Data for Expanded View [file EMBJ-39-e104948-s010.zip › EMBOJ-2020-104948R_SourceDataEV3.pdf]

**A**

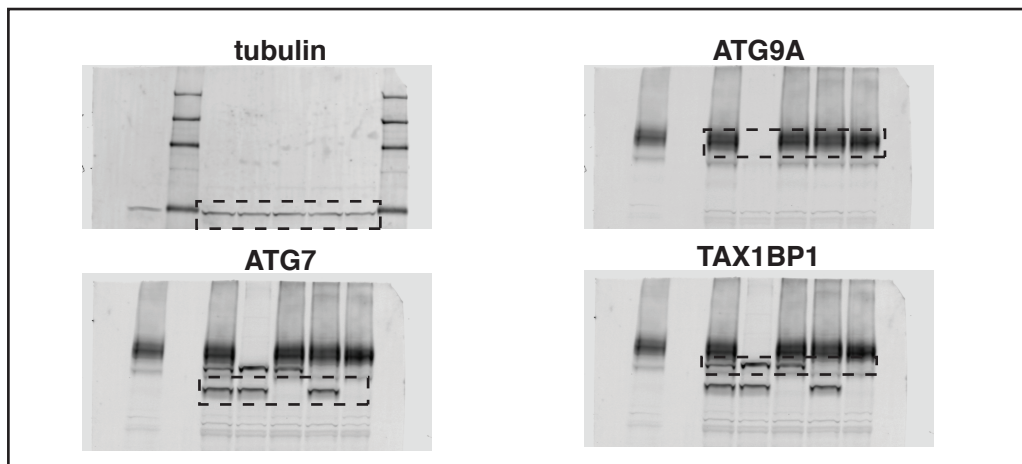

**F**

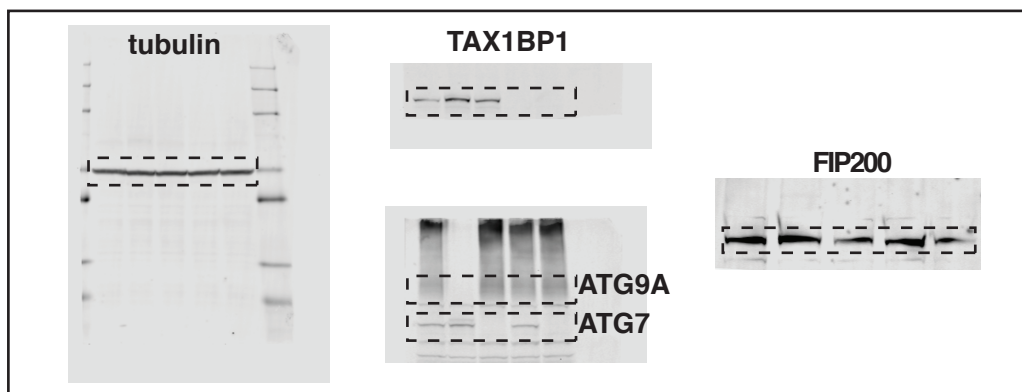

**H**

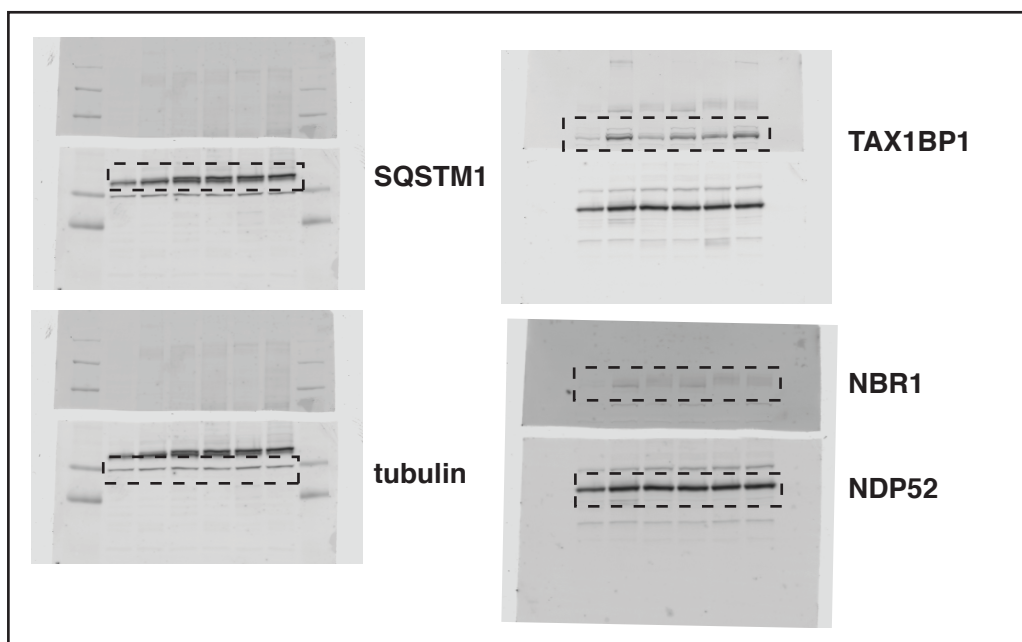

Supplement: Supplementary file 6 — Source Data for Expanded View [file EMBJ-39-e104948-s010.zip › EMBOJ-2020-104948R_SourceDataEV2.pdf]

**A**

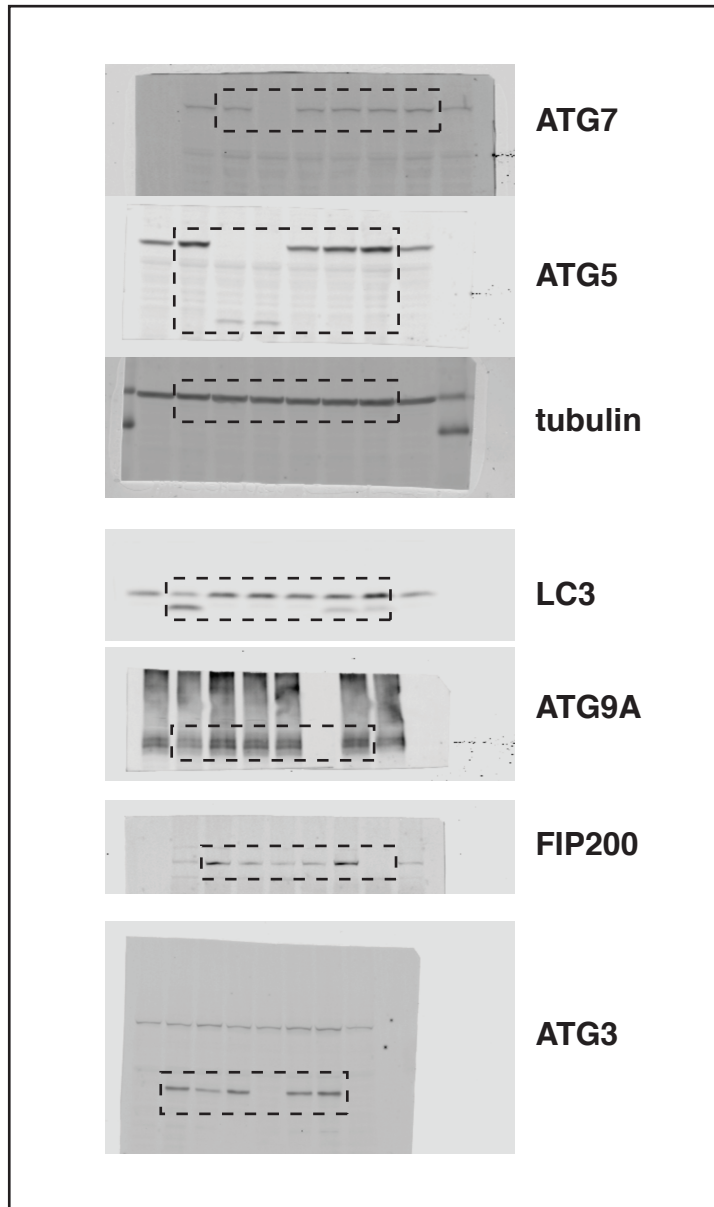

Supplement: Supplementary file 6 — Source Data for Expanded View [file EMBJ-39-e104948-s010.zip › EMBOJ-2020-104948R_SourceDataEV1.pdf]

**B**

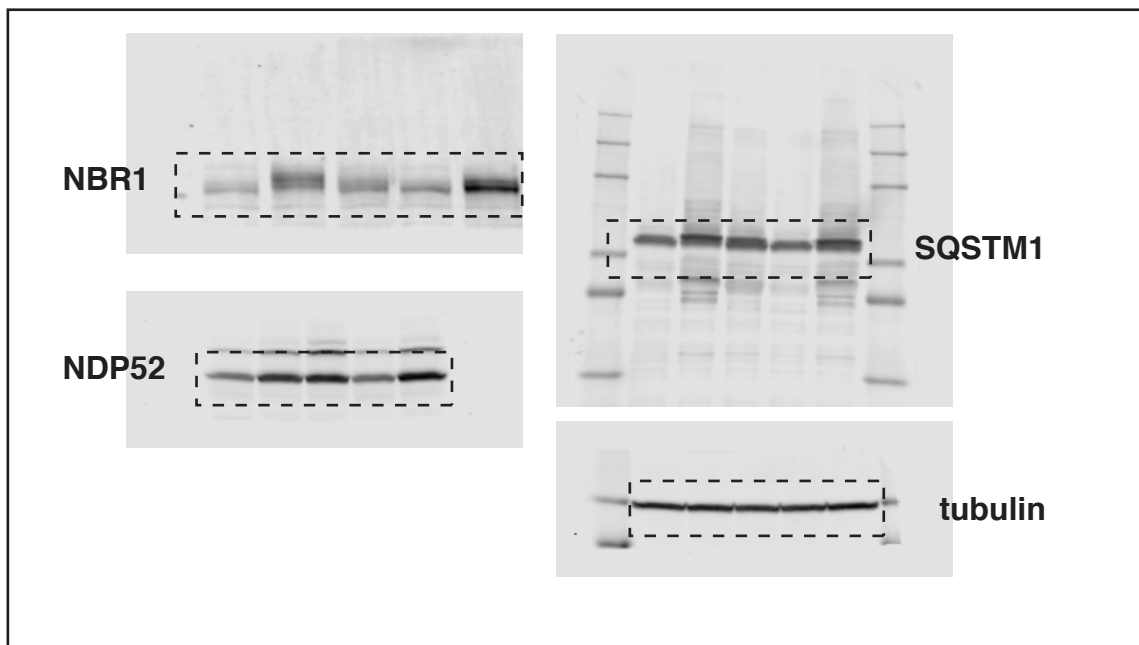

**D**

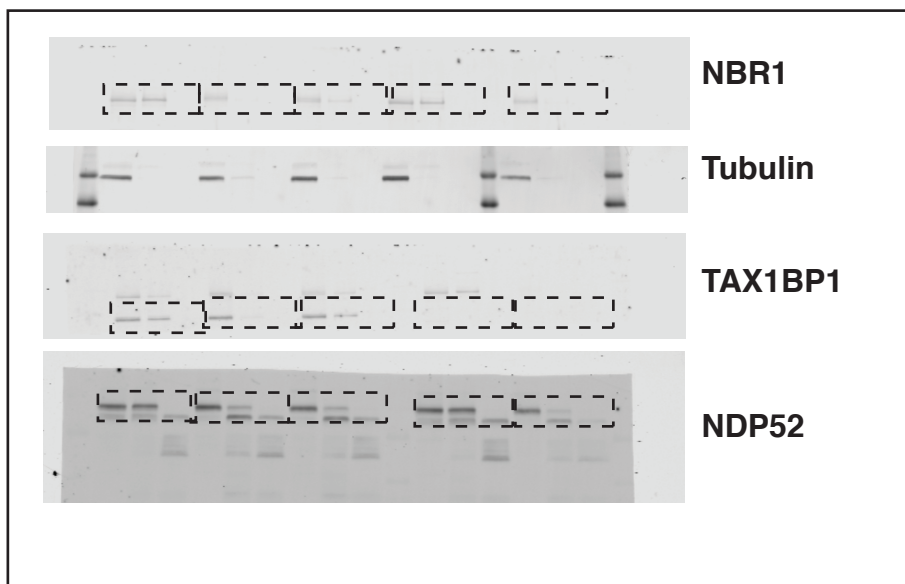

Supplement: Supplementary file 8 — Source Data for Figure 4 [file EMBJ-39-e104948-s006.pdf]

# Source data Figure 6

## C - TAX1BP1 panel

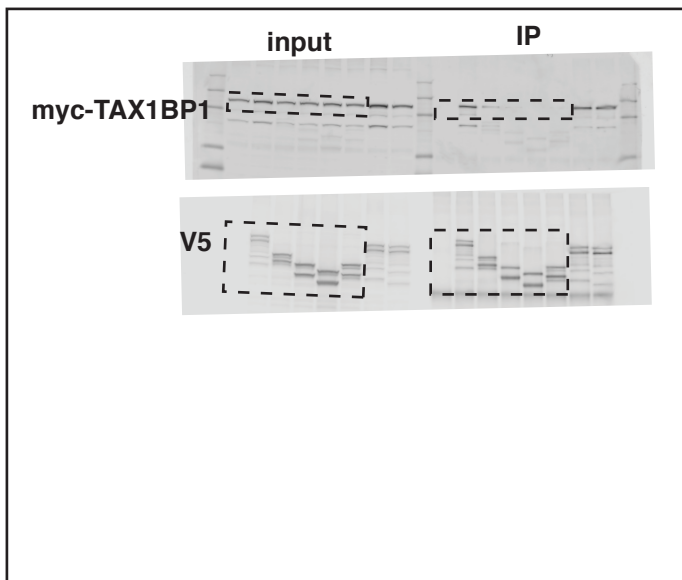

## C - NBR1 panel

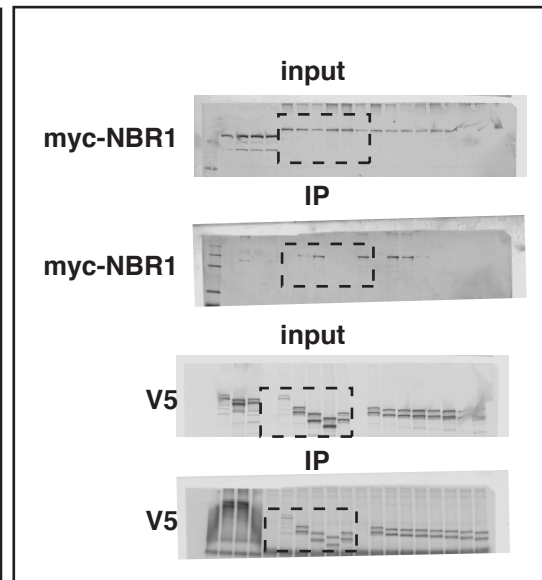

## D

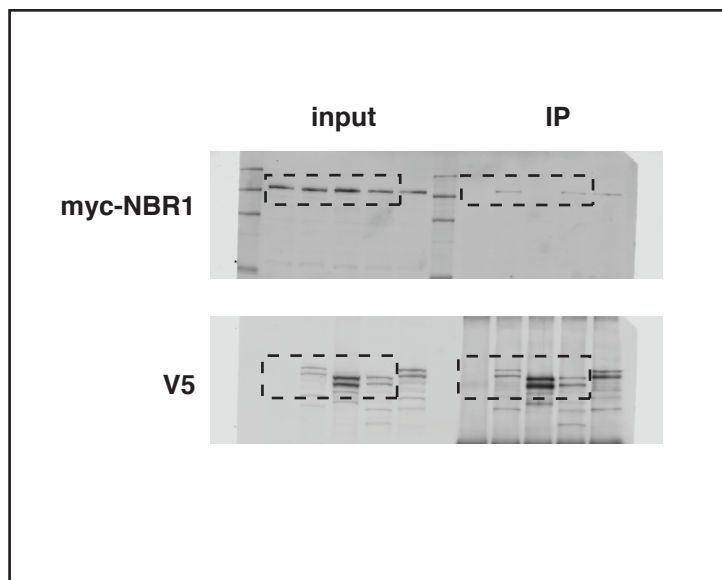

Supplement: Supplementary file 10 — Source Data for Figure 6 [file EMBJ-39-e104948-s008.pdf]
