# Supplementary material for: Receptor‐mediated clustering of FIP200 bypasses the role of LC3 lipidation in autophagy
Source: EMBO J. 2020 Nov 23;39(24):e104948. doi: 10.15252/embj.2020104948 (PMC7737610; doi:10.15252/embj.2020104948)

Source data Figure 5

Input samples

TAX1BP1

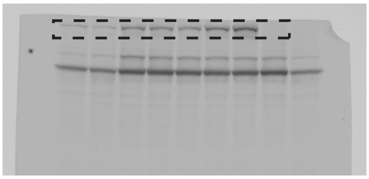

NBR1

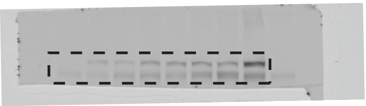

SQSTM1

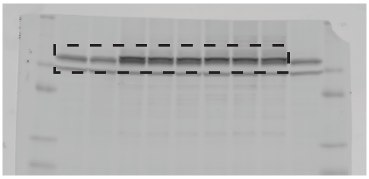

Tubulin

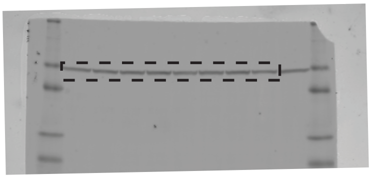

NDP52

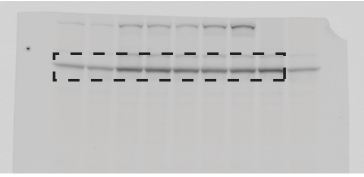

IP samples

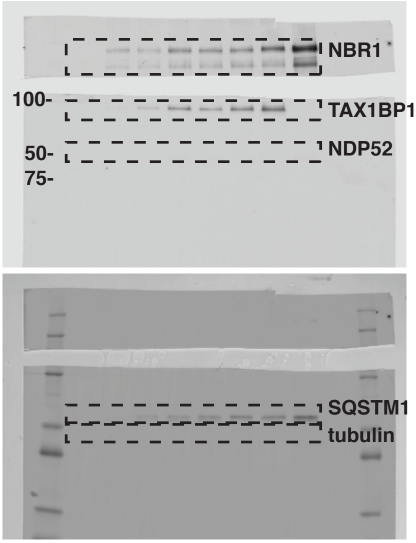

Supplement: Supplementary file 9 — Source Data for Figure 5 [file EMBJ-39-e104948-s007.pdf]
